# A pilot study to evaluate the feasibility of individualized yoga for inpatient children receiving intensive chemotherapy

**DOI:** 10.1186/s12906-015-0529-3

**Published:** 2015-01-24

**Authors:** Caroline Diorio, Tal Schechter, Michelle Lee, Cathy O’Sullivan, Tanya Hesser, Deborah Tomlinson, Janine Piscione, Christine Armstrong, George Tomlinson, Lillian Sung

**Affiliations:** Child Health Evaluative Sciences, The Hospital for Sick Children, Peter Gilgan Centre for Research and Learning, 686 Bay St, Toronto, ON M5G 0A4, Canada; Division of Haematology/Oncology, The Hospital for Sick Children, 555 University Ave, Toronto, ON M5G 1X8, Canada; Toronto General Hospital, 200 Elizabeth Street, Toronto, ON M5G 2C4, Canada

**Keywords:** Pilot, Yoga, Children, Chemotherapy, Haematopoietic stem cell transplantation, Fatigue, Quality of life

## Abstract

**Background:**

Fatigue is an important problem in paediatric cancer patients and yoga may be an effective intervention. The primary objective was to determine the feasibility of individualized yoga for hospitalized children receiving intensive chemotherapy.

**Methods:**

We included English-speaking children and adolescents aged 7–18 years receiving intensive chemotherapy or haematopoietic stem cell transplantation (HSCT). Yoga was conducted three times weekly for three weeks. The primary outcome was feasibility, defined as ability to deliver at least 60% of planned sessions. Secondary outcomes were parent-reported Pediatric Quality of Life Inventory (PedsQL) Multidimensional Fatigue Scale, Fatigue Scale-Parent, PedsQL Generic Core Scales and PedsQL Acute Cancer Module.

**Results:**

Between January and October 2013, 11 patients were enrolled. Median age was 14.0 (range 7.7-16.4) years and 6 (55%) were boys. Yoga was feasible with 10/11 participants meeting the threshold for feasibility. The median number of yoga sessions was 9 (range 3–13). No adverse events were attributed to yoga. Mean ± standard deviation for the day 21 proxy-reported PedsQL general fatigue scores was 55.6 ± 15.5. Qualitative comments suggested design changes for future yoga studies.

**Conclusions:**

Individualized yoga is feasible for inpatient children receiving intensive chemotherapy. Future work will include development and conduct of a randomized trial for fatigue amelioration.

**Trial registration:**

ClinicalTrials.gov NCT02105389.

## Background

Cancer-related fatigue can be described as a subjective feeling of physical, emotional, and/or cognitive tiredness [1]. It is thought to be commonly under-recognized, not reported and poorly managed [2]. Further, it is prevalent in children with cancer [3-5]. Three cross-sectional studies conducted in children 10 to 18 years of age found that lack of energy was the most common symptom, occurring in 50% to 76% of participants [4,6,7]; fatigue symptoms were moderate to very severe in intensity. Fatigue is frequently identified as the most troublesome symptom in paediatric cancer patients with an important impact on quality of life (QoL) [8]. Inpatient status and recent chemotherapy are significant predictors of worse fatigue in children [7,9]. Based on severity of illness, anaemia, concurrent medications, inactivity and prolonged inpatient status, children with cancer receiving intensive chemotherapies are expected to be at high risk for severe fatigue.

Guidelines suggest that all cancer patients, including children as young as 5 years of age, should be routinely screened for fatigue [10]. These guidelines also suggest fatigue should be managed according to clinical practice standards. However, evidence demonstrating effective interventions for fatigue in children with cancer is scarce. In a recent systematic review of 72 randomized controlled trials (RCTs) which included participants of all ages, exercise reduced fatigue by a moderate amount with a standardized mean difference of −0.45, 95% confidence interval (CI) -0.57 to −0.32 (P < 0.001). Type of exercise (aerobic, walking, resistance, yoga or mixed) did not impact on exercise efficacy (P for interaction = 0.35) [11]. Unfortunately, only one study was paediatric-specific.

We hypothesized that yoga would be an ideal intervention for children receiving the most intensive chemotherapy as these children may be too ill to participate in other types of exercise programs. Yoga is a unique intervention that combines exercise and mindfulness. Intensity of the yoga practice can be titrated to a patient’s current status and yoga can be delivered in any location without the need for specialized equipment.

In order to evaluate the efficacy of individualized yoga, a RCT is required. However, prior to proceeding to a definitive randomized trial, conduct of a pilot study is important to ensure that a RCT is feasible, to facilitate design of the RCT and to estimate variability of the outcome measure [12]. The primary objective was to determine the feasibility of individualized yoga for hospitalized children receiving intensive chemotherapy. Secondary objectives were to describe: (1) Parent/guardian proxy-report child fatigue scores; (2) Proxy-report child QoL; (3) Self-report parent QoL; (4) Number of children willing to self-report symptoms; and (5) Qualitative suggestions to improve trial conduct.

## Methods

This was a pilot trial of individualized yoga for fatigue in children 7 to 18 years of age receiving intensive chemotherapy at The Hospital for Sick Children (SickKids), Toronto. This study received Research Ethics Board approval from SickKids and all participants/guardians provided informed consent and assent as appropriate.

### Subjects

We included children and adolescents who were: (1) Diagnosed with any acute myeloid leukemia (AML), relapsed acute lymphoblastic leukemia, stage 3 or 4 Burkitt’s lymphoma/leukemia or about to receive autologous or allogeneic haematopoietic stem cell transplantation (HSCT); (2) Expected to be an inpatient for at least three weeks after initiation of chemotherapy or conditioning; and (3) Ages 7 to 18 years at enrollment. Exclusion criteria were as follows: (1) Features present to an extent that would preclude compliance with yoga (as assessed by the attending physician): a) motor disability, b) cognitive disability, c) cardiopulmonary symptoms, or d) known compression fracture resulting in disability; and (2) Parent or patient cannot understand English.

### Site

All subjects were recruited from a single site, SickKids in Toronto, Canada. SickKids is the tertiary care referral center for the Greater Toronto area and is the only center that performs paediatric HSCT in the province of Ontario.

### Study procedures

All participants received yoga; yoga began between day −1 to day +4 after starting any course of chemotherapy or conditioning regimens for HSCT. Treatment was administered by one of three trained children’s yoga instructors. All yoga instructors were certified teachers in children’s yoga and also received study-specific yoga training. It was intended for yoga to occur three times weekly for 3 weeks. However, mid-way through the pilot study, we appreciated that it was difficult to identify 3 appropriate days for yoga in advance because of acuity of illness, scheduling of tests such as computerized tomography scans and other radiological evaluations, and procedures. Consequently, we began to offer yoga daily excluding weekends and holidays (in other words, 4 to 5 times weekly) with the goal of achieving sessions three times weekly.

There was a common structure for all sessions that started with relaxation and breathing followed by a series of poses focused on strengthening, flexibility, and balance. Each session terminated with a period of relaxation (*savasana*). Yoga instructors were asked to select appropriate poses from a pre-determined list of poses for each session. There were low, moderate and high intensity regimens available depending on the wishes and abilities of the child and parent and the judgment of the yoga instructor. The target intensity was documented and could change with each yoga session. Children were encouraged to choose a focus for each session such as working on a new pose they learned in a previous session, practicing balancing, focusing on deep breathing or focusing on clearing their mind. For each session, the parent or another family member was offered the opportunity to perform yoga along with the child and instructor.

### Outcomes

The primary outcome was feasibility, defined as the ability to deliver at least 60% of the planned three times weekly yoga sessions in at least 70% of participants. The time frame evaluated began at the first session and extended until day 21, discharge date or date off study, whichever occurred first. For secondary outcomes, parent proxy-reported child fatigue and QoL were of main interest. We also included child self-reported fatigue and QoL as well as parent self-reported QoL to identify the feasibility and utility of these measures. Parents were the primary respondents while children were encouraged to self-report if they wished. All instruments used a 7 day recall period and were administered at baseline and on days 7, 14 and 21 except the Short Form-36 (SF-36) which was administered at baseline and on day 21.

In order to measure fatigue, we included the two instruments with sufficient psychometric evaluation to permit inclusion in trials [13], namely the Pediatric Quality of Life Inventory (PedsQL) Multidimensional Fatigue Scale (MFS) [14] and the Fatigue Scale-Child, Adolescent and Parent (FS-C, FS-A and FS-P) [15]. The PedsQL MFS is an 18-item instrument that assesses general fatigue, sleep/rest fatigue and cognitive fatigue; it is reliable and valid in children with cancer [14]. We were most interested in general and sleep/rest fatigue. The FS-P is proxy-report and the FS-C (10-items) and FS-A (13-items) are child and adolescent self-report. All scales are reliable, valid and sensitive to change when used longitudinally [16,17].

To measure child QoL, we used the PedsQL 4.0 Generic Core Scale and PedsQL 3.0 Acute Cancer Module. The PedsQL is a multidimensional instrument that is reliable and valid in healthy populations and in children with cancer [14,18-21]. From the generic scale, we were most interested in the physical and psychosocial summary scores. From the cancer module, we were most interested in pain and hurt, nausea, anxiety and worry.

In order to measure parent QoL, the Short Form (SF)-36 was used. The SF-36 is composed of 36 items that measure physical function, role physical function, bodily pain, general health perception, mental health, role emotional function, energy, and social function [22,23]. Scores for each domain range from 0 to 100, with higher scores indicating better QoL. It results in a physical and a mental health component summary score which have a mean of 50 and standard deviation of 10, representing the mean and standard deviation of the general United States population. The SF-36 is valid and internally consistent [22,23].

In addition to these quantitative measures, qualitative comments were collected at each assessment time point throughout the study. Participants were asked what they liked and disliked about the yoga program and whether or not they felt the yoga program was of benefit.

### Statistical analysis

Feasibility was defined as the ability to deliver at least 60% of the planned three times weekly yoga sessions over the evaluation time frame in at least 70% of participants. We had planned to enroll a minimum of 10 and a maximum of 20 patients to allow modifying the program in the event significant barriers were identified.

## Results and discussion

Between January and October 2013, we assessed 22 potential patients. Of those screened, 5 refused, 6 were missed (not identified during the period of eligibility) and 11 consented to participate. Demographic characteristics are illustrated in Table 1. Median age was 14.0 (range 7.7-16.4) years and 6 (55%) were boys. Six children had AML (3 with acute promyelocytic leukemia) and 6 (55%) were undergoing HSCT. Two children who participated had co-morbidities. One child had ataxia and a compression fracture (not resulting in disability) and one child had cellulitis.

**Table 1:** **Demographics of the study cohort**

**Characteristic**	**Value N** = **11**
Male (%)	6 (54.6)
Median age in years (range)	14.0 (7.7-16.4)
Diagnosis (%)	
Leukemia/lymphoma	8 (72.7)
Solid tumor	1 (9.1)
Brain tumor	1 (9.1)
Aplastic anemia	1 (9.1)
Median months since diagnosis (IQR)	2.2 (0.2, 8.6)
Relapse status (%)	1 (9.1)
Current treatment	
Chemotherapy (%)	5 (45.4)
Stem cell transplantation (%)	6 (54.6)
Parent/other participated in at least one session (%)	9 (81.8)
Child previous experience with yoga	3 (27.3)

We found that yoga was feasible with 10/11 participants meeting the *a priori* defined threshold. The median number of yoga sessions was 9 (range 3 to 13). Only one 7 year old child stopped yoga after three sessions because it was not “fast paced” enough for her. No adverse events were experienced, and specifically no musculoskeletal, bleeding or central line issues were attributed to yoga. When the total number of sessions was considered, 26% were associated with family-member participation. Of the 11 participants, 8 had a family member participate in at least one session. The median age of children who had at least one session with a family member co-participating was 13.5 (range 7 to 16) years. The ages of the child participants without any family member participation were 13, 14 and 16 years.

The proxy-report fatigue scores according to the PedsQL MFS and FS-P are illustrated in Table 2. Proxy-report generic and cancer-specific QoL are also illustrated in Table 2. Only 3 children self-reported fatigue and QoL scores and thus, these scores are not presented. Parent/guardian self-reported QoL according to the SF-36 is illustrated in Table 3.

**Table 2:** Proxy report fatigue and quality of life scores*

		**Day 0**		**Day 7**		**Day 14**		**Day 21**
	**N**	**Median (IQR)**	**N**	**Median (IQR)**	**N**	**Median (IQR)**	**N**	**Median (IQR)**
**Fatigue**								
*PedsQL Multi*-*dimensional Fatigue Scale*								
General fatigue	11	50.0 (20.8, 70.8)	10	56.3 (35, 62.5)	9	50.0 (33.3, 70.8)	9	50.0 (45.8, 70.8)
Sleep/Rest Fatigue	11	62.5 (20.8, 66.7)	10	41.7 (29.2, 54.2)	9	33.3 (29.2, 41.7)	9	54.2 (37.5, 66.7)
*Fatigue Scale* - *Parent*	10	40.0 (34.0, 44.0)	10	46.5 (44.0, 52.0)	10	45.5 (39.0, 49.0)	9	44.0 (43.0, 48.0)
**Quality of Life**								
*PedsQL Cancer Module*								
Pain and hurt	11	50.0 (37.5, 75.0)	10	62.5 (25.0, 87.5)	7	50.0 (25.0, 75.0)	9	75.0 (37.5, 75.0)
Nausea	11	50.0 (35.0, 75.0)	9	50.0 (35.0, 55.0)	7	60.0 (35.0, 80.0)	9	50.0 (40.0, 75.0)
Procedural Anxiety	11	50.0 (25.0, 91.7)	9	58.3 (25.0, 100)	6	95.8 (50.0, 100)	9	66.7 (41.7, 100)
Treatment Anxiety	11	75.0 (50.0, 100)	9	66.7 (50.0, 100)	6	87.5 (50.0, 100)	9	75.0 (75.0, 100)
Worry	11	58.3 (41.7, 83.3)	10	62.5 (50.0, 83.3)	7	75.0 (50.0, 83.3)	9	75.0 (50.0, 83.3)
*PedsQL Generic Core*								
Physical health	11	64.3 (40.6, 71.9)	10	48.4 (12.5, 68.8)	8	59.1 (34.4, 73.4)	6	59.9 (25.0, 75.0)
Psychosocial health	11	68.8 (60.0, 81.7)	10	62.8 (56.7, 70.5)	8	73.8 (65.3, 79.6)	9	70.0 (65.0, 80.0)

Abbreviation: *IQR* = interquartile range *Where N < 11, evaluations were missing.

**Table 3:** Parent quality of life scores*

		**Day 0**		**Day 21**
	**N**	**Median (IQR)**	**N**	**Median (IQR)**
Physical Health Summary	9	47.7 (45.0, 62.6)	7	52.2 (41.5, 61.0)
Physical functioning	11	46.5 (40.2, 57.0)	8	52.8 (42.3, 57.0)
Role-physical	10	47.1 (37.3, 56.9)	7	54.4 (32.4, 56.9)
Bodily pain	10	53.3 (41.4, 62.1)	8	58.8 (41.4, 62.1)
General health	10	45.8 (38.6, 57.7)	8	56.5 (45.8, 62.0)
Mental Health Summary	9	38.4 (33.8, 45.9)	7	48.1 (29.5, 56.7)
Vitality	11	49.0 (33.4, 58.3)	8	44.3 (38.1, 59.9)
Social functioning	11	45.9 (24.1, 51.4)	8	48.7 (35.0, 56.8)
Role-emotional	10	42.3 (36.4, 55.9)	8	44.3 (26.8, 55.9)
Mental health	10	44.4 (33.1, 50.0)	8	50.0 (38.7, 54.2)

Abbreviation: *IQR* = interquartile range *Where N < 11, evaluations were missing.

Qualitative feedback from both children and parents indicated physical and psychological benefits of yoga. Both children and parents noted physical impacts such as increased energy levels, decreased nausea and a reduced need for pain medication. Psychological benefits included reduced anxiety and agitation, better sleep and improved mood. Many children felt yoga had given them the opportunity to relax and ‘escape’ from the hectic hospital environment.

Several comments will lead to design changes as follows. First, parents felt that four assessments were overly burdensome and that there were too many questionnaires with each assessment. Second, parents noted the social and school functioning questions of the PedsQL Generic Core Scales were not relevant given the inpatient status of their children. Finally, we found that three times weekly yoga was not an optimal schedule since children may not be available for yoga on a given day related to illness or medical procedures/diagnostic tests. We asked the last 3 children enrolled whether they would participate in a future RCT of yoga if given the opportunity; all 3 said they would participate.

We found that individualized yoga in children receiving intensive chemotherapy is feasible. Our patient population primarily consisted of children with AML and those undergoing HSCT. These children are expected to become profoundly neutropaenic with multiple toxicities of therapy including fever, invasive infection and mucositis and a relatively high treatment-related mortality rate. Consequently, the ability to conduct yoga in this intensively treated and often acutely ill population is very encouraging. We also found that it is feasible to measure fatigue and QoL in these children provided parents are the primary respondents.

In addition, our study provided insight into ways to improve the design of a future RCT. Because of the burdensome nature of outcome assessments, we will reduce the number of assessments to three: baseline, day 10 and day 21. We plan to eliminate the PedsQL Generic Core Scales because of lack of relevance and the SF-36 since caregiver health is not of primary interest, in order to decrease the burden of questionnaire administration. Finally, to improve the ability to deliver yoga, we will change the intervention schedule to daily excluding weekends. Our data also support proxy-response as the primary outcome since very few children were willing to self-report symptoms.

Our results are concordant with three other yoga feasibility studies. First, Thygeson and colleagues [24] explored the feasibility of a single yoga session for children 7 to 18 years hospitalized with cancer or other blood disorders. A yoga class was held in the inpatient unit playroom and administered by a researcher who was also a registered yoga teacher. Eleven children participated; the session was feasible and received positive feedback. A second feasibility study of 5 weekly yoga sessions was conducted in 6 children with a variety of cancer types [25]. In this study, a low-impact program was developed by the authors that incorporated elements such as stretching, strengthening and relaxation. The yoga sessions were one hour long, conducted in a group setting, were uniquely adapted to individual patients and were facilitated by physical and occupational therapists, assistants and rehabilitation aides [25]. One child with AML (5 years of age) and two children post HSCT (12 and 19 years of age) were included. The intervention was feasible; children found that they looked forward to participating in the yoga sessions and that the sessions always made them feel better afterwards [25]. A third study demonstrated the feasibility of a 12 week community based program for out-patient children with any type of cancer. Sessions were conducted twice weekly and the authors reported preliminary evidence of program benefits on QoL [26]. Our study is unique, however, as it applies individualized yoga up to 5 times weekly among an intensively treated cancer and HSCT inpatient paediatric population and evaluated the feasibility of measuring specified fatigue and QoL outcomes.

Because of the single armed nature of this pilot study, it is not possible to estimate the effect of yoga on fatigue and QoL outcomes and thus, the definitive RCT will be important to clarify the utility of yoga in this setting. The ability to translate the intervention to a multi-center trial will rely upon careful description of the yoga intervention and training. In our feasibility study, we developed and followed a detailed yoga program and plan to disseminate this information in a subsequent publication. For the future RCT, we plan to provide in-person training to site yoga instructors and to monitor 20% of yoga sessions remotely to ensure fidelity to the program and safety of the intervention. The future RCT will need to consider the minimally clinically important difference in the fatigue outcome in order to appropriately power the study.

The strengths of this study are the *a priori* designated definition of feasibility and identification of several key ways to improve the design of the future RCT. However, there are limitations of our study. The most important limitation is that we did not perform randomization within this pilot study and thus, we did not demonstrate the feasibility of enrolling children to a randomized trial. However, all 3 participants who were asked, stated that they would participate in a future RCT of yoga if invited, which provides some reassurance. Second, we performed this pilot study at a single site to ensure we had maximum control of the sessions and outcome ascertainment while we were still in the development phase of the yoga program. A third limitation was the defined threshold for feasibility, namely completion of at least 60% of the planned three times weekly sessions. This threshold was chosen based on balancing what was likely to be achievable in such an intensively treated population at risk for life-threatening complications and the number of sessions expected to be required to achieve benefit. However, we recognize that this threshold is lower than standards in the exercise field. Another limitation is that we did not define other elements of feasibility, such as duration of sessions, intensity of sessions and completion of required poses. Further research should focus on such elements.

## Conclusions

In summary, individualized yoga is feasible for children with cancer receiving intensive chemotherapy and undergoing HSCT. Future work will proceed toward development and conduct of a RCT of individualized yoga to reduce fatigue in children with cancer and HSCT. Identification of an effective intervention is an important step toward reducing fatigue and improving QoL in this patient population.

## Abbreviations

AML: Acute myeloid leukemia
CRF: Cancer-related fatigue
FS-A: Fatigue scale-adolescent
FS-C: Fatigue scale-child
FS-P: Fatigue scale-parent
HSCT: Haematopoietic stem cell transplantation
MFS: Multidimensional Fatigue Scale
PedsQL: Pediatric Quality of Life Inventory
QoL: Quality of Life
RCT: Randomized Controlled Trial
